# A Bearing Fault Diagnosis Model Based on a Simplified Wide Convolutional Neural Network and Random Forrest

**DOI:** 10.3390/s25030752

**Published:** 2025-01-26

**Authors:** Qikai Zhang, Yunan Yao, Yage Huang, Yangbowen Liu, Linfeng Wu

**Affiliations:** School of Naval and Power Engineering, Wuhan University of Technology, Wuhan 430070, China; zqk_13@163.com (Q.Z.); 18740563321@139.com (Y.H.); 2865600779@139.com (Y.L.); wulf729@whut.edu.cn (L.W.)

**Keywords:** fault diagnosis, deep learning, ball bearings, vibration signal

## Abstract

Bearings play a crucial role in the complex mechanical systems of ships, and their operational status is closely related to vibration signals. Therefore, analyzing bearing signals plays an important role in the field of fault diagnosis. In order to solve the problems of low accuracy and slow response speed in fault diagnosis through vibration signals at mixed speeds, this paper introduces an improved Simple Window Deep Convolutional Neural Network with Random Forest (SWDCNN-RF) model on traditional Wide Convolutional Neural Network (WDCNN). It was verified through the publicly available dataset of ball bearings from Western Reserve University in the United States. It was found that the improved model increased speed by 38.51% and accuracy from 97.5% to 99.6% at epoch = 50, and also achieved faster convergence and smaller fluctuations during training. This study is of great significance for determining the occurrence time and type of bearing faults, and provides criteria for reliability evaluation and fault diagnosis of equipment using bearings.

## 1. Introduction

Maritime transportation plays a very important role in today’s economic development, and the reliability of complex mechanical systems on board ships is particularly important. With the rapid development of science and technology, fault diagnosis is an important part of reliability assessment. Traditionally, fault diagnosis of maritime machinery is carried out through physical inspection, sensor-based monitoring, and the expertise of experienced maritime engineers. The recent advent of digital technologies and the emergence of big data have opened up new avenues for innovation in maritime machinery fault diagnosis [1]. Any malfunction within these bearings has the potential to cause a complete mechanical system failure, which could lead to catastrophic accidents [2]. The health and performance of rotating machinery heavily rely on the condition of the bearings that support the rotating shaft. The condition of bearings is significantly impacted by their inherent nonlinearity, causing nonlinear vibrations that affect machinery health and performance [3]. Therefore, fault diagnosis technology for rolling bearings holds significant importance in the industrial sector, and the research on bearing fault diagnosis has been booming over the past years [4]. The fault diagnosis process for ball bearings involves collecting vibration signals and extracting fault characteristics. Initially, this involves techniques such as acoustics [5], electrical current [6], acoustic emission [7], and vibration [8]. Vibration signals, being the longest-standing and most effective method, are widely employed to detect abnormalities in rolling bearings [9,10]. By attaching acceleration sensors to the bearing seat, the vibration characteristics of rolling bearings can be accurately and efficiently measured.

The development of fault diagnosis technology has evolved from traditional signal processing methods to modern machine learning and deep learning methods. Traditional methods such as time-domain analysis, frequency-domain analysis, and time-frequency analysis can initially identify the type and extent of faults by extracting and analyzing the characteristics of vibration signals. With the advancement of computing power and data processing technology, machine learning methods such as support vector machines, decision trees, and random forests, as well as deep learning methods such as convolutional neural networks (CNNs), are increasingly being used in fault diagnosis. These methods can automatically extract complex features from a large amount of vibration data, improving the accuracy and efficiency of fault diagnosis. CNN can be widely used in many fields of machine learning algorithms, and its core is image recognition. Nowadays, more and more research shows the role of the CNN algorithm in fault diagnosis. So far, there are two main directions for the application of CNNs in fault diagnosis. One is to use an image sensor to capture image data of diagnostic targets on a regular basis, such as explicit images, temperature values, and so on. This method is commonly used in engineering in the direction of power systems; another more common method is to treat CNN as a feature extractor, combined with data preprocessing and other auxiliary networks for fault diagnosis [11]. For example, Variational Mode Decomposition (VMD) is used to decompose the original bearing vibration signal into a series of modal components containing fault features, and CNN is used to extract the fault features, while Long Short-Term Memory (LSTM) is used to extract the time series information of the fault signal [12]. As in reference [13], a gray texture image-based intelligent fault diagnosis method was proposed. It converts vibration signals into images and uses a CNN with Adaptive Batch Normalization (AdaBN) to enhance diagnostic accuracy and generalization under variable load conditions. A new correlation analysis algorithm for motion steady-state signals and collected signals is proposed in reference [14], which combines CNN with FFT for motion aging. In reference [15], a novel intelligent diagnosis method for rolling bearing and rotor composite faults based on vibration signal-to-image mapping and the combination of Convolutional Neural Networks (CNNs) and Support Vector Machines (SVMs) was proposed. As in reference [16], the original vibration of the training bearing is first processed using the Hilbert-Huang transform to construct a new nonlinear degradation energy index. Then, CNN technology is used to identify and extract the difference between the extracted degradation energy index and the original vibration of the trained bearing. Hidden mode, used to predict the degradation life of bearings. It can be seen that CNNs combined with preprocessed raw data or other deep learning techniques can complete fault diagnosis [17]. Therefore, it can be seen that the current mainstream research basically uses vibration signals to study single faults, while there is still much room for development in using speed signals for fault diagnosis. As in reference [18], a deep learning algorithm that takes into account the motor speed is proposed in the literature. The vibration signal is regarded as an image, and then the CNN algorithm is used for fault diagnosis. In recent years, with the continuous progress of science and technology, data-driven fault diagnosis methods based on machine learning have gradually become mainstream in the field of fault diagnosis [19].

However, unlike 2D and 3D CNNs, which are widely used in image recognition, 1D CNNs that process one-dimensional vibration data can lead to a structure that is too deep if all stacked small convolution kernels. To address this issue, an improved deep CNN model with a wide first layer kernel (WDCNN) was proposed and successfully applied to fault diagnosis [20]. The first layer uses a wide convolution kernel, while other layers use a series of small kernels, which can achieve a balance between structure and feature extraction [21]. The Random Forest (RF) classifier is one of the most powerful classification algorithms [22]. The main idea behind the RF algorithm is to use a combination of randomized trees and predict by majority vote among all generated decision trees [23]. This in turn provides high accuracy and robustness in dealing with different problems by reducing the variance of classification and achieving lower generalization error [24]. This article will combine WDCNN and random forest and simplify the structure of WDCNN to achieve the purpose of rolling bearing fault diagnosis.

## 2. SWDCNN-RF Basic Theory

The fault diagnosis process can be broken into three key stages: signal acquisition, feature extraction, and fault pattern recognition. Traditional approaches to fault diagnosis, while commonly used, tend to be slow, less accurate, and require a high degree of precision for data classification. These limitations make them less effective for diagnosing real-world rolling bearing faults.

The WDCNN is a deep learning model designed specifically for one-dimensional data processing. It utilizes wider convolutional kernels and larger strides to efficiently capture broad, significant features from input signals. This is particularly useful for extracting meaningful patterns from one-dimensional vibration signals. By combining wide kernels and large strides, WDCNN not only enhances the model’s ability to capture local features, but it also reduces the number of convolutional layers and overall parameters. As a result, the model becomes computationally more efficient. The incorporation of batch normalization and pooling layers further accelerates the training process, improves stability, and reduces both data dimensionality and computational complexity, all of which help to mitigate the risk of overfitting.

Building on this foundation, this paper introduces an optimized model called Simple Window Deep Convolutional Neural Network with Random Forest (SWDCNN-RF), which merges a simplified version of the WDCNN with Random Forest (RF) for enhanced fault diagnosis. In this approach, the original WDCNN’s five-layer convolutional structure is simplified to just two layers. To compensate for any potential loss of information due to this reduction, frequency domain features are introduced, unlike the original model, which relied solely on time-series data. This addition of frequency domain data increases the richness of the input information and helps reduce noise, improving the model’s generalization ability. Compared to WDCNN, SWDCNN has fewer layers, which simplifies the model and can reduce training time. In addition to temporal data, SWDCNN also incorporates frequency domain features. With a smaller number of parameters, SWDCNN requires fewer computational and storage resources, making it a suitable choice for environments with limited resources.

In addition, an encoder layer is incorporated to extract critical features from the input data, which may include both one-dimensional features and rotational speed. These refined features are then passed into a Random Forest algorithm. Compared to traditional convolutional neural networks, Random Forest provides superior classification performance and faster computational speed.

RF has better generalization performance and robustness compared to other single algorithms and is suitable for various types of data and task scenarios. The steps of the algorithm are as follows:

(1) Select a training set containing N samples, with each sample including M features;
(2) Randomly select k features (k < M) as the candidate set of features for node splitting in each decision tree;
(3) For each node, select the optimal feature from the candidate feature set for splitting to minimize the impurity (Gini index or information entropy) after the split;
(4) Repeat steps 2 and 3 to construct n decision trees;
(5) For a new test sample, input it into each decision tree and obtain a comprehensive prediction through methods such as averaging or voting.

**Figure 1:**
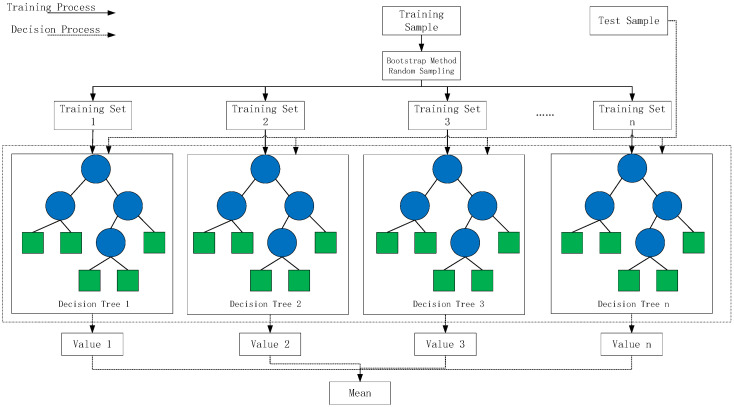
Training diagram of the Random Forest algorithm.

The training diagram of the Random Forest algorithm is shown in Figure 1. In the diagram, blue nodes represent the root and internal nodes, and green nodes indicate leaf nodes that can no longer be split. During the prediction process, the Random Forest calculates the sample values, and the final value is obtained by summing the values of each tree and dividing by the number of trees.

By combining the feature extraction power of SWDCNN with the classification accuracy of Random Forest, the SWDCNN-RF model achieves a highly efficient and accurate diagnostic solution.

**Figure 2:**
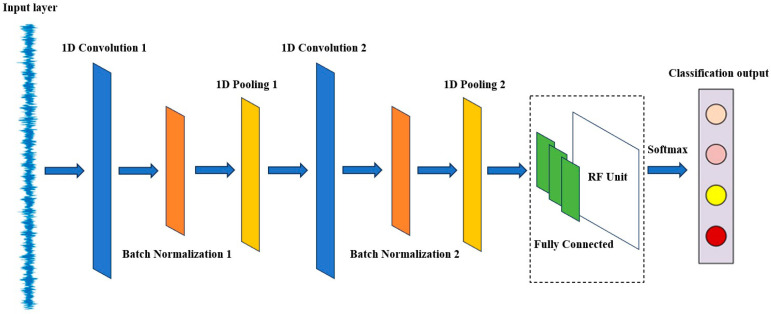
Schematic diagram of model structure.

The proposed rolling bearing fault diagnosis model, based on the Simple Wide Convolutional Neural Network (SWDCNN), is depicted in Figure 2. This model consists of two wide convolutional layers, two max-pooling layers, a batch normalization layer, a dropout layer, two fully connected layers, an encoder layer, a Random Forest classifier, and a Softmax output layer. This architecture ensures computational efficiency while maintaining high diagnostic accuracy, making it well suited for practical industrial applications.

The first convolutional layer employs a wide convolutional kernel (size 64) with a large stride (16), enabling it to capture a broad spectrum of features in the input vibration signal. This layer is followed by a Batch Normalization layer and a ReLU activation function, which together speed up the training process and enhance model stability. Next, a max pooling layer (with a pooling window size of 2) is applied to reduce data dimensionality and computational complexity. The second convolutional layer uses a smaller kernel size of 3, with a stride of 1, and the input data are appropriately padded. This layer also includes a batch normalization layer and a ReLU activation function, followed by another max pooling layer to further decrease data dimensionality. After these convolutional and pooling operations, the features are flattened into one-dimensional vectors, and a Dropout layer (with a dropout rate of 0.2) is applied to prevent overfitting. The model then passes through two fully connected layers—the first with 32 neurons and the second serving as an encoder, reducing feature dimensionality to 8 neurons. The output layer utilizes a Softmax activation function to convert the encoder layer’s output into a class probability distribution for fault classification.

Convolutional neural networks (CNNs), a fundamental aspect of deep learning, excel in handling both one-dimensional and multidimensional data, enabling the automatic extraction of features from raw data to complete recognition tasks. In the context of rolling bearing fault diagnosis, employing the SWDCNN-RF model eliminates the need for manual feature extraction, thereby significantly improving fault classification accuracy. This chapter provides an overview of the SWDCNN structure and the foundational principles of Random Forest (RF) before explaining how the SWDCNN-RF model is constructed to address the challenges of processing mixed data under varying operational conditions. This combined approach enhances diagnostic accuracy, reduces fault detection time, and boosts overall efficiency.

In processing one-dimensional vibration signals, the convolutional layer is a critical component of SWDCNN. By utilizing wide convolutional kernels and larger strides, this layer captures a broad range of features from the input signal, allowing for a reduction in the number of layers and parameters, which in turn improves computational efficiency. The convolutional operations focus on extracting local signal features, such as frequency components and amplitude changes, which are key to effective feature extraction in fault diagnosis tasks. This method ensures that critical signal characteristics are identified and used to improve fault recognition, making the diagnostic process faster and more accurate.

**Figure 3:**
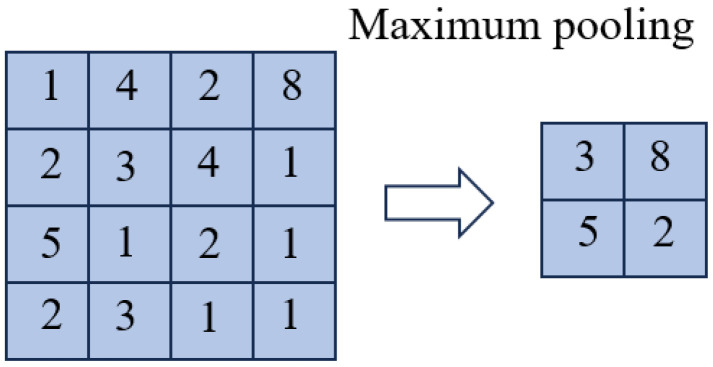
Diagram of maximum pooling operation.

Typically following the convolutional layer, the pooling layer reduces data dimensionality and computational complexity. Max pooling is used within this layer to extract the maximum value within each pooling window, retaining the most critical features and minimizing the risk of overfitting. Additionally, the pooling layer improves the translation invariance of features, which is particularly important when processing vibration signals, as fault features may shift along the time axis. Figure 3 illustrates the max pooling process.

After the pooling operation, the size of the feature map is (M, Fs), where Fs is the number of filters in the layer and M is calculated by the following formula:

(1) $$M=\frac{W-F}{S}+1$$

In the formula, W represents the length and width of the input image, F represents the size of the convolution kernel, and S represents the moving step size.

The batch normalization layer is applied after the convolutional layer to standardize the input for each subsequent layer. By implementing batch normalization, SWDCNN can speed up the training process while enhancing the model’s stability and performance. This layer helps address the issue of internal covariate shift, ensuring that the input distribution for each layer remains relatively stable. As a result, the model converges more quickly and achieves higher accuracy. Batch normalization not only boosts the training efficiency but also strengthens the network’s generalization ability. The specific steps of batch normalization are as follows:

(1) Calculate the mean μ and variance $\sigma^{2}$ of the current small batch;
  (2) $$\mu =\frac{1}{m}\sum_{i=1}^{m}x_{i}$$
  (3) $$\sigma^{2}=\frac{1}{m}\sum_{i=1}^{m}{\left(x_{i}-\mu \right)}^{2}$$
(2) Calculate the standardized $x_{i}^{\prime }$, subtract the mean μ of the small batch from the input, and then divide by the standard deviation $\sqrt{\sigma^{2}+\varepsilon }$, where ε is a very small constant to prevent the phenomenon of dividing by 0;
  (4) $$x_{i}^{\prime }=\frac{x_{i}-\mu }{\sqrt{\sigma^{2}+\varepsilon }}$$
(3) Enlarge $x_{i}^{\prime }$, multiply by the same scaling value $\gamma_{i}$, and add a bias value $\beta_{i}$ to enhance the network expression ability after batch normalization layer.
  (5) $$y_{i}=\gamma_{i}\times x_{i}^{\prime }+\beta_{i}$$

After the convolution and pooling operations, the extracted signal features are flattened into one-dimensional vectors. These vectors are then fed into a fully connected layer, which further refines and combines the features using linear transformations and nonlinear activation functions like ReLU. In SWDCNN, multiple fully connected layers are typically employed, each containing a different number of neurons. This layered architecture allows the model to capture more complex, higher-level features, enhancing its ability to represent intricate patterns within the data. The fully connected layers work synergistically with the Softmax classifier, which is responsible for translating the extracted and refined features into distinct categories. By combining the feature extraction capabilities of the convolutional and pooling layers with the decision-making power of the fully connected layers and the Softmax classifier, SWDCNN achieves a high level of classification accuracy and expressive power, enabling it to handle complex tasks with greater precision.

**Figure 4:**
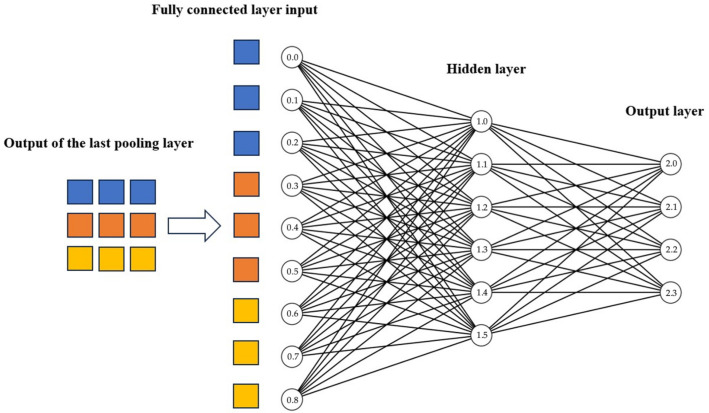
Schematic diagram of the fully connected layer.

The output of the final pooling layer is flattened into a one-dimensional feature vector, which is then fed into the fully connected layer, where it is fully connected to both the input and output. The output layer utilizes the Softmax function to convert the input neurons into a probability distribution, summing to 1, which predicts the likelihood of each bearing state. The forward propagation calculation formula for the fully connected layer is as follows:

(6) $$z^{l+1\left(j\right)}=\sum_{i=1}^{n}W_{ij}^{l}\alpha^{l\left(i\right)}+b_{j}^{l}$$

where $z^{l+1}$ is the logits value of the *j*-th neuron in the (*l* + 1)-th layer, and $W_{ij}^{l}$ is the weight between the *i*-th neuron in the first layer and the *j*-th neuron in the (*l* + 1)-th layer, $b_{j}^{l}$ is the bias value of all neurons in the first layer to the *j*-th neuron in layer (*l* + 1)-th. Figure 4 is a schematic diagram of the fully connected layer.

The expression for the ReLU function is

(7) $$f\left(x\right)=\left\{\begin{array}{c} x,x>0\\ 0,x\leq 0 \end{array}\right.=\max\{0,x\}$$

**Figure 5:**
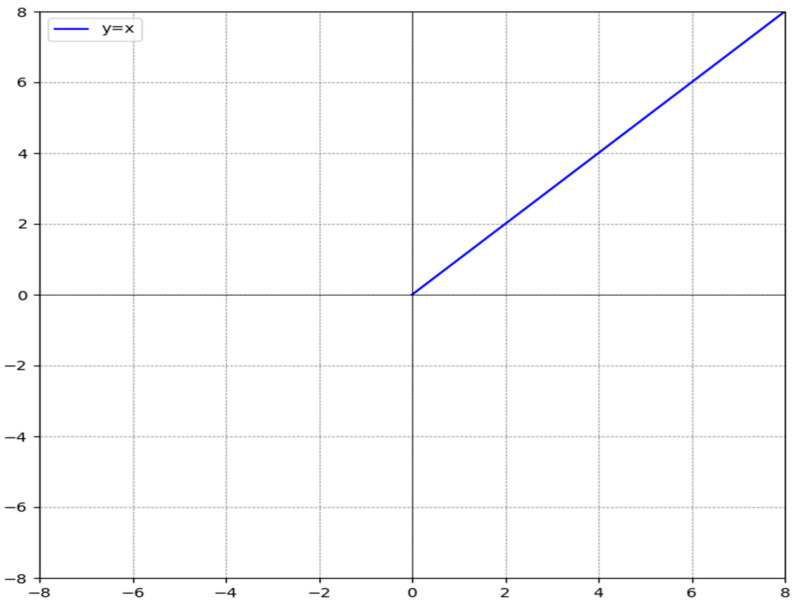
ReLU activation function.

A curve graph based on the function expression is drawn, as shown in Figure 5. From Figure 4, it can be seen that when the input is less than or equal to 0, the output of the ReLU function remains constant at 0. When the input is greater than 0, the output value is equal to the input value, that is, when the input is a positive number, the derivative of the ReLU function is 1, and the gradient vanishing problem is solved.

Precisely because the ReLU function’s uniqueness lies in its ability to effectively address the gradient vanishing problem, it accelerates the training process and improves model stability in the convolutional layers mentioned earlier. Therefore, it is necessary to use Figure 4 to intuitively understand the working principle of the ReLU function and its response characteristics to positive input values. Although the function itself seems simple, Figure 4 is indispensable for enhancing the understanding of the ReLU function and illustrating its role in deep learning.

The primary role of the encoder layer is to reduce the complexity of high-dimensional feature vectors by distilling the most relevant features for further analysis, especially when paired with Random Forest (RF) models. In SWDCNN, this layer typically utilizes fewer neurons (for instance, reducing from 32 to 8 neurons) to create a more streamlined model architecture and decrease the computational load. This reduction is achieved by applying linear transformations along with activation functions like ReLU, which map the high-dimensional features into a more manageable, lower-dimensional space. This simplification not only facilitates subsequent tasks like classification or regression but also enhances processing speed and efficiency.

Random Forests, on the other hand, are adept at optimizing their performance by automatically fine-tuning hyperparameters during training, ensuring the best possible classification results. When combined with the encoder layer, the SWDCNN-RF model excels in both computational efficiency and improved generalization. This synergy significantly elevates classification accuracy while minimizing the risk of overfitting.

As the final component of the model, the output layer plays a crucial role in delivering the ultimate predictions or classifications. In SWDCNN, the output layer typically uses a Softmax activation function to transform the encoder’s output into a probability distribution over the potential classes. For binary classification tasks, this layer consists of two neurons, each representing the likelihood of one of the two possible outcomes. In multi-class classification, the number of neurons in the output layer corresponds to the number of categories, enabling the model to produce accurate predictions for each class.

## 3. Fault Diagnosis of Rolling Bearings Based on SWDCNN-RF

### 3.1. Dataset Introduction

To verify the feasibility of the proposed model, the bearing dataset from Western Reserve University in the United States will be used for feasibility validation [25].

**Figure 6:**
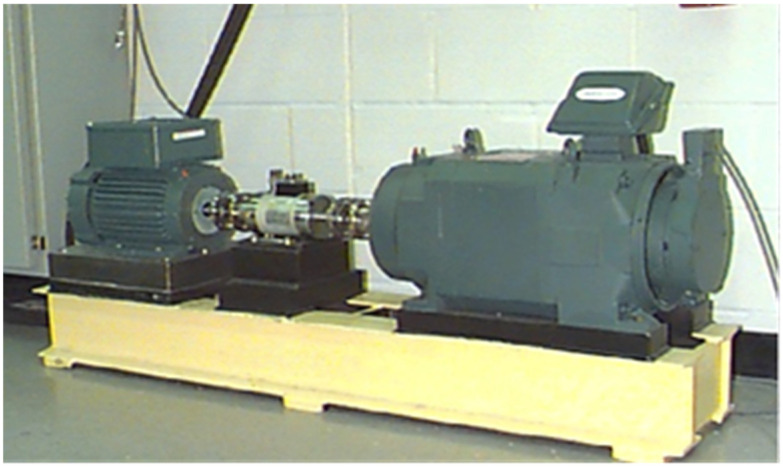
Rolling bearing fault simulation test bench.

The experimental setup for diagnosing faults in rolling bearings, as shown in Figure 6, utilizes a test bench provided by Case Western Reserve University. This bench is equipped with accelerometers to monitor vibration signals, and the bearings tested are 6205-2RJEM SKF deep groove ball bearings. The sampling frequency of the setup is 48 kHz, adhering to the sampling theorem’s requirement that the sampling frequency must be at least twice the maximum frequency of the signal to ensure accurate data capture. This ensures that the obtained data are reliable and suitable for analysis, meeting the necessary experimental standards.

The experiment was conducted at four different rotational speeds: 1797 r/min, 1772 r/min, 1750 r/min, and 1730 r/min. To simulate various fault conditions, fault diameters of 0.007 inches, 0.014 inches, and 0.021 inches were introduced. The study evaluated four operating conditions: inner ring fault, rolling element fault, outer ring fault, and normal condition. These conditions were used for both validation and testing. The data were split into training and test sets, with a ratio of 7:3, ensuring an adequate dataset for model training and performance evaluation.

**Figure 7:**
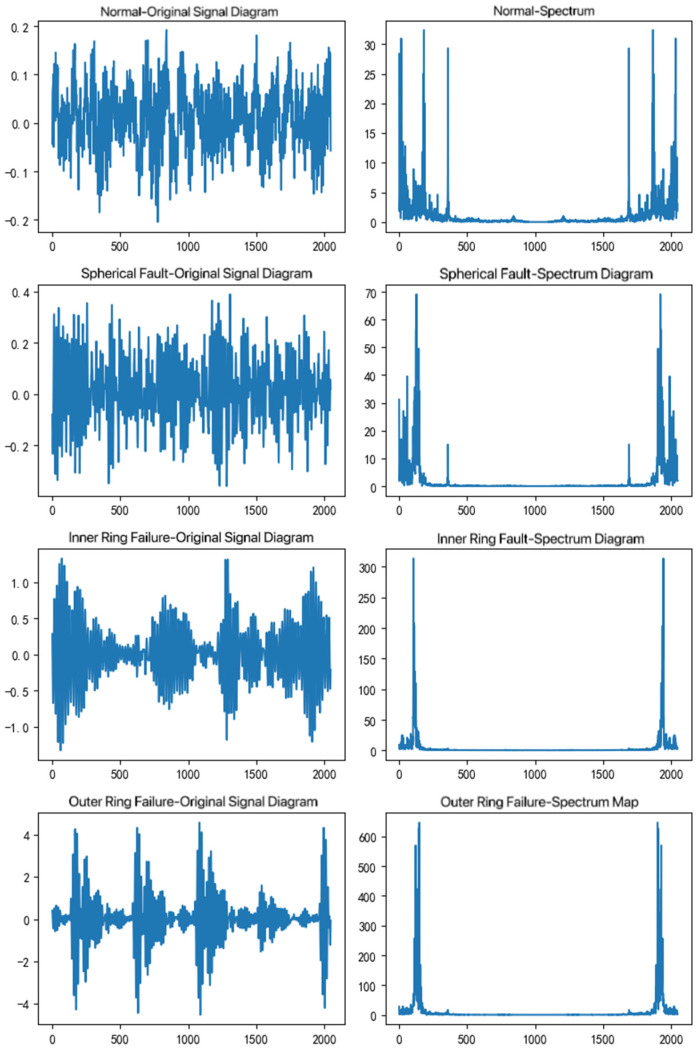
Fault signal and spectrum diagram.

By analyzing the vibration data at these four speeds, the signal and frequency spectra of the original normal, spherical fault, inner ring fault, and outer ring fault can be obtained, as shown in Figure 7. The axis title of this figure is amplitude.

### 3.2. Application of Methods

**Figure 8:**
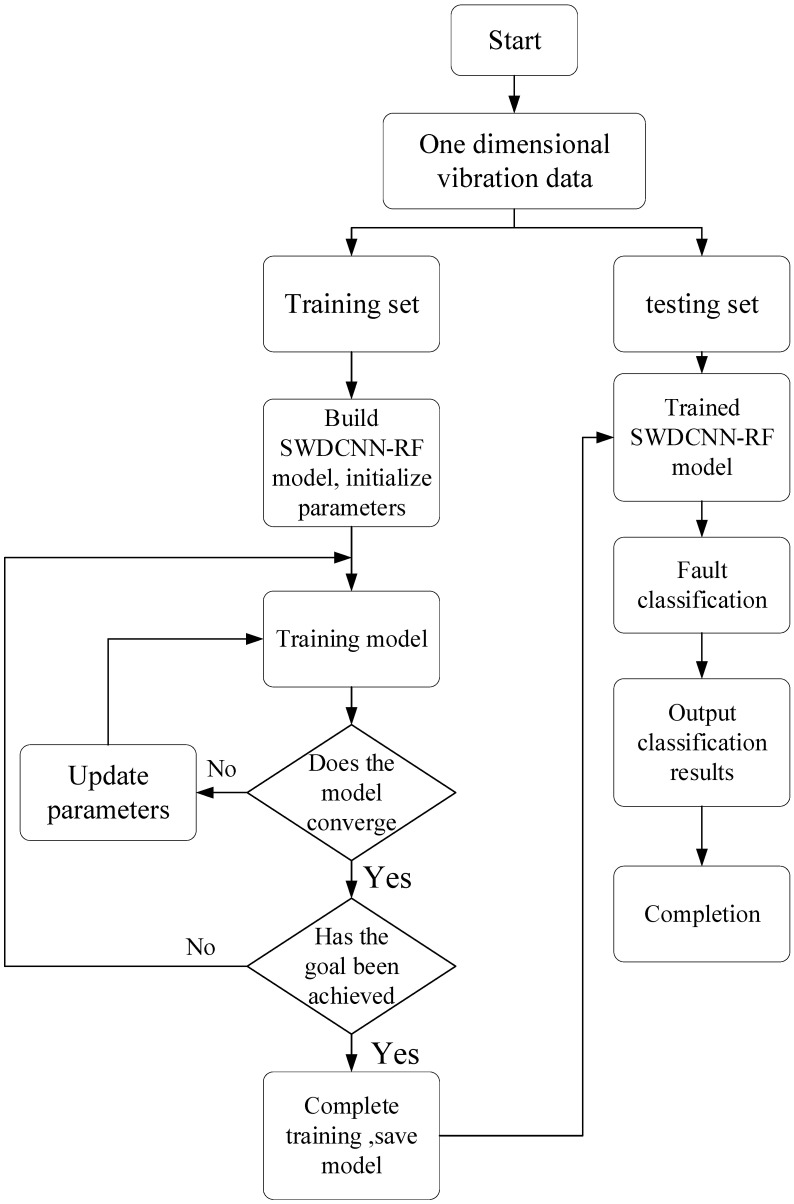
Diagnostic process.

Figure 8 shows the fault diagnosis flowchart of the model proposed in this chapter. Firstly, the training set is input into the built model for training. When the model reaches the set expected goals, the test set is input into the model for fault diagnosis, and the model outputs the diagnostic results of the test set.

Firstly, the CWRU rolling bearing dataset is preprocessed, including the labeling of fault modes, the combination of fault data at different rotational speeds, and the denoising of vibration data. Next, as previously mentioned, the dataset is divided into training and testing sets at a ratio of 7:3.

For the training set, the SWDCNN model is established, and parameters are initialized. The model initialization includes the name, input shape, and number of classes as parameters. During initialization, placeholders for the loss function, optimizer, and evaluation metrics are defined. The model can take one-dimensional signals with an input shape of (1, 1024). The first convolutional layer transforms the input into 16 channels with a kernel size of 64 and a stride of 16. The padding is calculated using ‘self.clac_padding’, resulting in an output shape of (16, 128). Batch normalization is then applied to the 16 channels, with both input and output shapes being (16, 128). The first activation layer uses the ReLU function. The first pooling layer has a window size of 2, halving the length. Thus, the input shape of (16, 128) is reduced to an output shape of (16, 64). The second convolutional layer takes the output from the first pooling layer, resulting in an output shape of (32, 64), with 32 channels and a kernel size of 3 and a stride of 1. Padding is again calculated using ‘self.clac_padding’. Following this, the second batch normalization layer normalizes the 32 channels, maintaining the shape (32, 64). The second activation layer also employs the ReLU function. The second pooling layer, with a window size of 2, further halves the length, changing the input shape from (32, 64) to an output shape of (32, 32). The flatten layer converts the convolutional output into a one-dimensional vector, resulting in an output shape of (1, 1024). Dropout is applied, randomly discarding 20% of the neurons to prevent overfitting. There are three fully connected layers: the first fully connected layer outputs 32 neurons, the second fully connected layer (encoder layer) outputs 8 neurons, and the third fully connected layer (output layer) produces neurons corresponding to the number of classes, with shapes of (1, 2) or (1, num_classes). The Softmax layer computes the probability distribution for each class using the Softmax function. The specific input and output sizes are detailed in Table 1. After constructing the model, training is conducted to observe convergence. If convergence is not achieved, model parameters are readjusted. Upon achieving convergence, the model’s performance is evaluated to ensure it meets the fault diagnosis requirements. If the requirements are not met, further parameter adjustments are made. Once the model satisfies the requirements, the test set is input, and the SWDCNN-RF model is trained to obtain fault modes and recognition results.

The implementation code is as follows:

Model initialization section:
class sWDCNN(BasicTorchModel):
  def __init__(self, name, input_shape=(1, 1024), num_classes=2):
   super().__init__()
   self.model_name = name
   self.loss_fn = None
   self.optimizer = None
   self.metric_fn = None
The first convolutional layer:
padding_1 = self.clac_padding(input_shape[1], 64, 16)
   self.conv1d_1 = nn.Conv1d(input_shape[0], 16, kernel_size=64, stride=16, padding=padding_1)
   self.bn_1 = nn.BatchNorm1d(16)
   self.active_1 = nn.ReLU()
   self.pool1d_1 = nn.MaxPool1d(2)
The second convolutional layer:
input_size_2 = int(np.floor(np.ceil(input_shape[1] / 16) / 2))
   padding_2 = self.clac_padding(input_size_2, 3, 1)
   self.conv1d_2 = nn.Conv1d(16, 32, kernel_size=3, stride=1, padding=padding_2)
   self.bn_2 = nn.BatchNorm1d(32)
   self.active_2 = nn.ReLU()
   self.pool1d_2 = nn.MaxPool1d(2, 2)
Flattening and fully connected layer:
self.flatten = nn.Flatten()
   self.dropout = nn.Dropout(0.2)
   input_size_3 = int(np.floor(input_size_2 / 2) * 32)
   self.linear_3 = nn.Linear(input_size_3, 32)
   self.active_3 = nn.ReLU()
Encoder and output layer:
self.linear_4 = nn.Linear(32, 8)
   self.active_4 = nn.ReLU()
   self.linear_5 = nn.Linear(8, num_classes)
   self.active_5 = nn.Softmax(dim = 1)

### 3.3. Experimental Comparative Analysis

This article will compare the SWDCNN-RF model, the traditional WDCNN model, and the CNN-ResNeSt model separately and use the three models to process the same dataset.

The traditional WDCNN model and the CNN-ResNeSt model were chosen as comparison models because WDCNN is a widely used deep learning model in the field of fault diagnosis, known for its powerful feature extraction capabilities and efficient processing of vibration signals. As a comparison model, it can clearly demonstrate the improvements of SWDCNN-RF in terms of speed and accuracy. On the other hand, CNN-ResNeSt is an advanced model that incorporates residual blocks and a multi-branch architecture. Using CNN-ResNeSt as a comparison model can illustrate that the improvements of SWDCNN-RF in speed and accuracy are even more significant compared to such an advanced model, allowing for a further evaluation of SWDCNN-RF’s performance. By comparing with these two models, the advantages of SWDCNN-RF can be more comprehensively demonstrated.

The specific parameters of the SWDCNN-RF model are shown in Table 2, the specific parameters of the traditional WDCNN model are shown in Table 3, and the specific parameters of the CNN-ResNeSt model are shown in Table 4 (with label num = 4).

**Figure 9:**
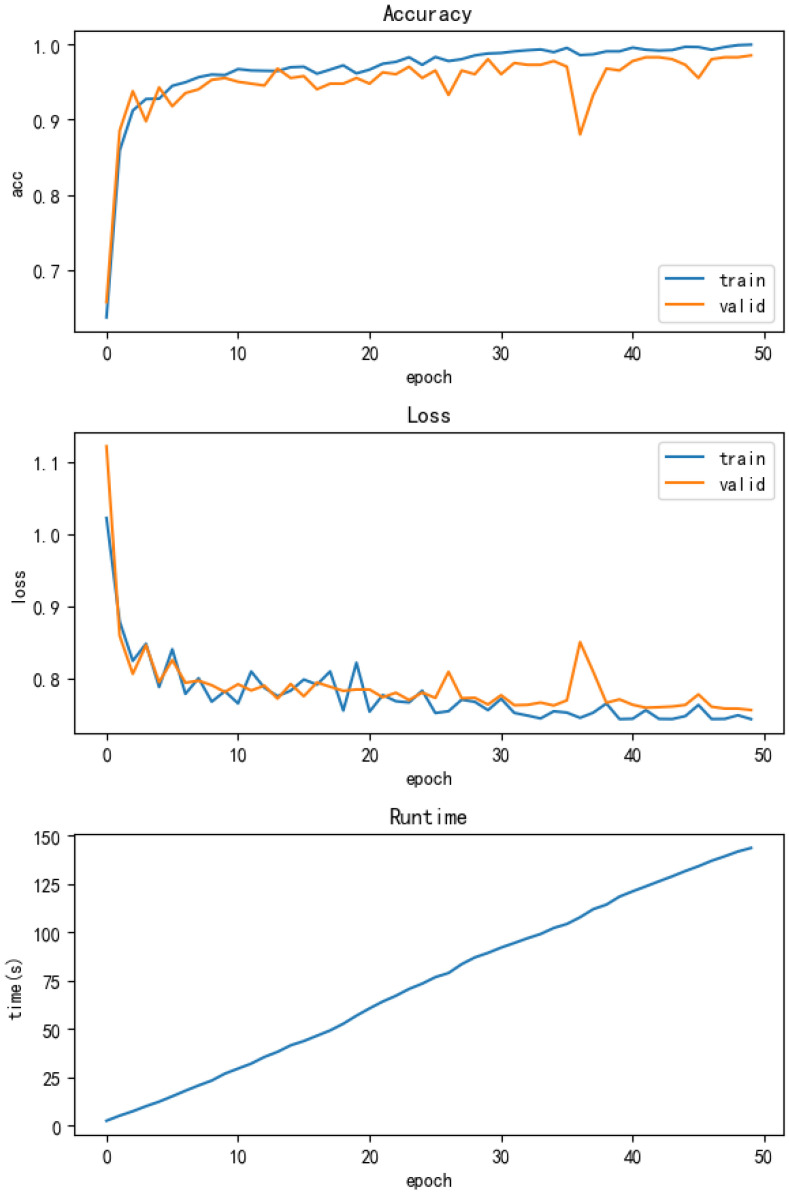
WDCNN accuracy and running time.

WDCNN is a relatively mature algorithm that performs well in fields such as fault diagnosis, image recognition, and speech processing. WDCNN, with its powerful feature extraction ability and good generalization performance, is widely used in the state monitoring and fault diagnosis of mechanical equipment. By processing data such as vibration signals and acoustic signals, WDCNN can accurately identify fault types and improve equipment operation reliability and maintenance efficiency. When we used the multi-condition dataset as input, we still achieved a good accuracy of 97.5% in the test set, demonstrating its strong adaptability and robustness. Upon closer observation of the training process, we can observe that although high accuracy was ultimately achieved, there was a certain degree of fluctuation in the training accuracy curve and loss curve. This fluctuation may be caused by the complexity and diversity of the data. And the training epoch is 50, taking 148 s, which is also too long. The accuracy and running time of the model are shown in Figure 9.

**Figure 10:**
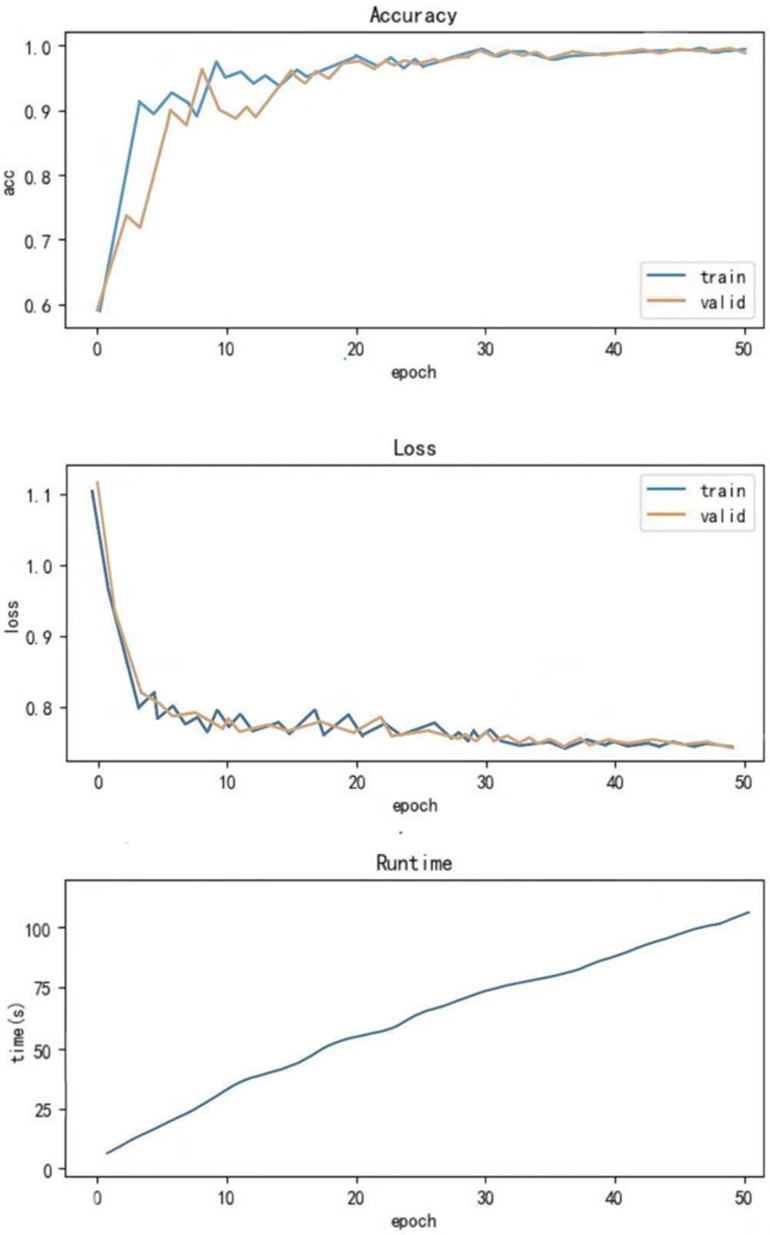
CNN-ResNeSt accuracy and running time.

Subsequently, the CNN-ResNeSt model was used for experiments. The CNN-ResNeSt model leverages a multi-branch architecture and attention mechanism, effectively enhancing feature extraction capabilities to capture both global and local features of bearing signals. On the experimental dataset, the CNN-ResNeSt model achieved a classification accuracy of 98.7%, slightly higher than the WDCNN model. Its training time was 123 s (epoch = 50), which is an improvement compared to WDCNN’s 148 s. The accuracy and running time of the model are shown in Figure 10.

We propose a new algorithm, SWDCNN-RF, which optimizes the original WDCNN by simplifying its structure. The original five-layer convolutional network has been reduced to two layers, streamlining the model and improving computational efficiency. To mitigate any potential information loss from this reduction, we introduced frequency domain features, whereas the original WDCNN relied solely on one-dimensional temporal data. By adding frequency domain features, the model’s information dimension is expanded, reducing noise interference and enhancing its generalization capability.

An encoder layer has also been incorporated to extract crucial features from the input data, including one-dimensional features such as rotational speed. These refined features are then processed by the Random Forest algorithm, which has a stronger classification ability than a CNN and offers faster computational speed. The integration of the encoder and Random Forest allows for more effective classification and faster model performance.

Since the SWDCNN-RF model is in the early stages of training, the incorporation of the random forest module increases the training complexity, and the training effectiveness of the random forest module affects the final output of the model. As the random forest module performs classification based on features extracted by the convolutional neural network, the entire feature fusion process requires more time to effectively function and optimize the weights of each module. In contrast, in the early epochs, WDCNN, as a purer convolutional neural network structure, is easier to converge, thus resulting in higher accuracy than SWDCNN-RF.

**Figure 11:**
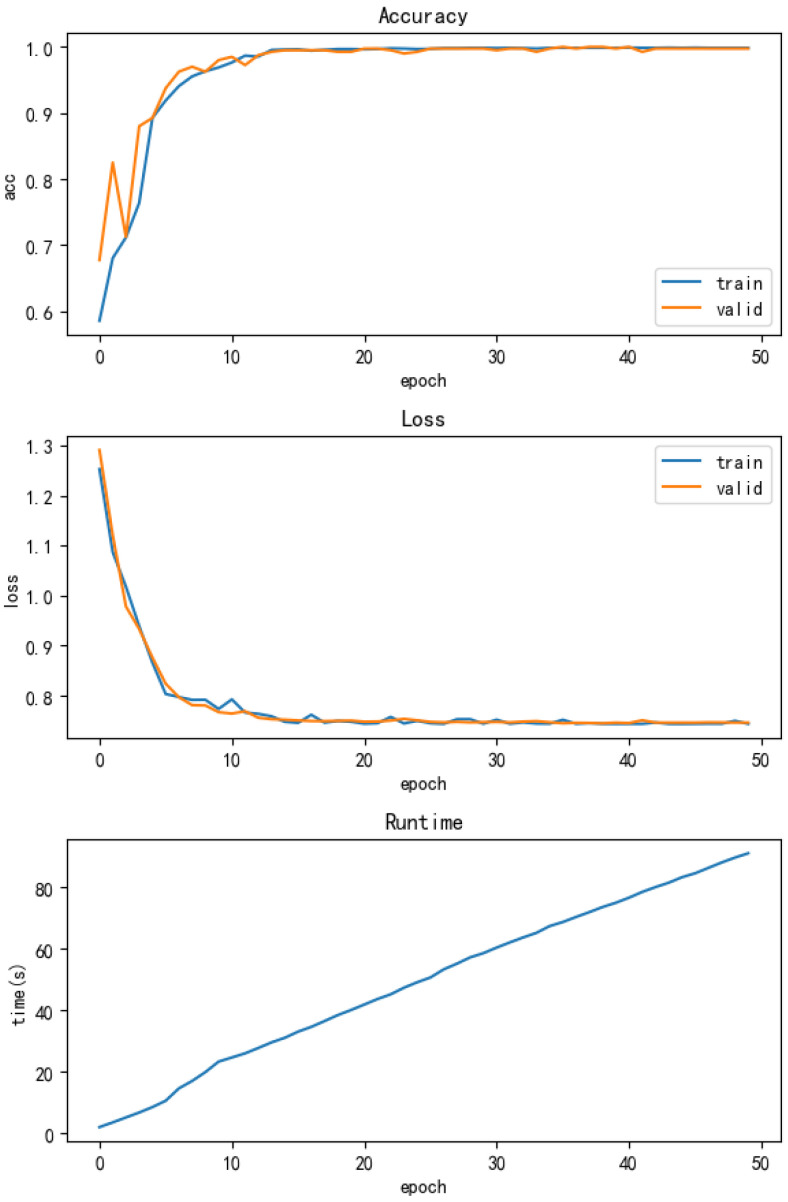
Accuracy and running time of SWDCNN-RF.

The training process for the SWDCNN-RF algorithm was conducted over 50 epochs, with a total training time of 91 s, representing a 38.51% reduction in time. The model’s accuracy also improved significantly, increasing from 97.5% to 99.6%, and convergence occurred more quickly during the training process. The enhanced performance and reduced computational time are depicted in Figure 11, which illustrates both the accuracy and running time improvements achieved with the SWDCNN-RF model.

**Figure 12:**
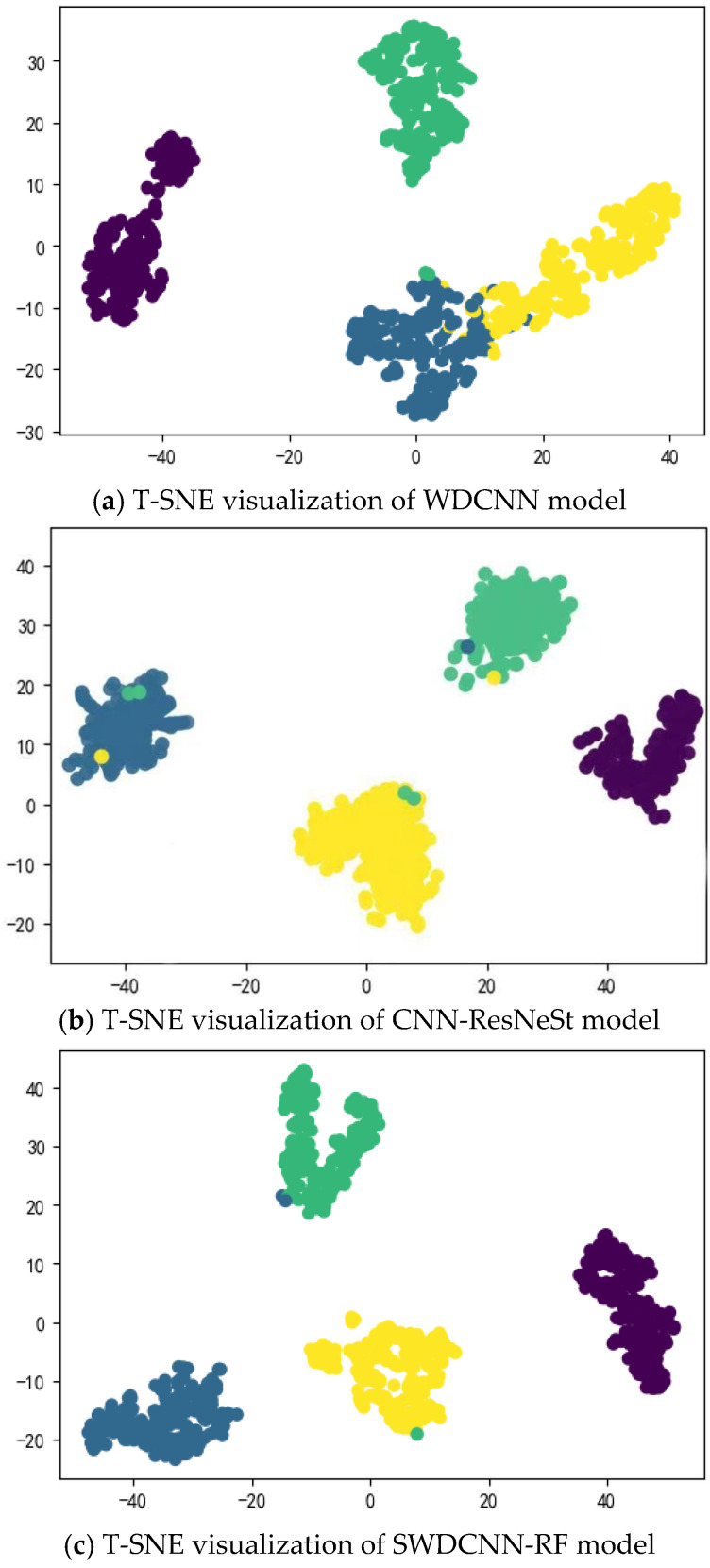
t-SNE visualization.

In addition, by observing the t-SNE visualization of the model outputs of the three algorithms, as shown in Figure 12, it is not difficult to see that SWDCNN-RF is clearer than WDCNN in the distance between the faults and normal conditions of the three types of ball bearings, and the confusion of the two fault modes will be less seen on SWDCNN-RF. SWDCNN-RF utilizes the ensemble learning characteristics of random forests to better capture complex features in data, increasing the robustness and generalization ability of the model. In contrast, traditional WDCNN models may have insufficient feature extraction or overfitting issues when dealing with different fault modes, resulting in unclear boundaries between different fault modes and increasing the confusion rate of classification. Through the t-SNE visualization method, we can intuitively see that while CNN-ResNeSt shows notable improvements compared to WDCNN, the distribution of SWDCNN-RF in the feature space, which can more effectively distinguish different types of fault states. This further verifies the superiority of SWDCNN-RF in handling ball bearing fault diagnosis problems. This improvement not only significantly improves accuracy but also demonstrates stronger advantages in the interpretability and practicality of the model, especially in practical industrial applications, providing higher guarantees for the accuracy and reliability of fault diagnosis.

**Figure 13:**
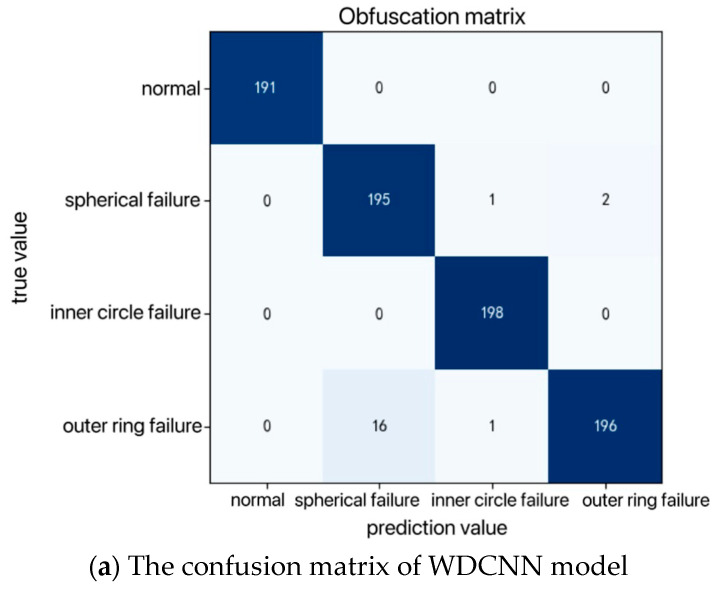

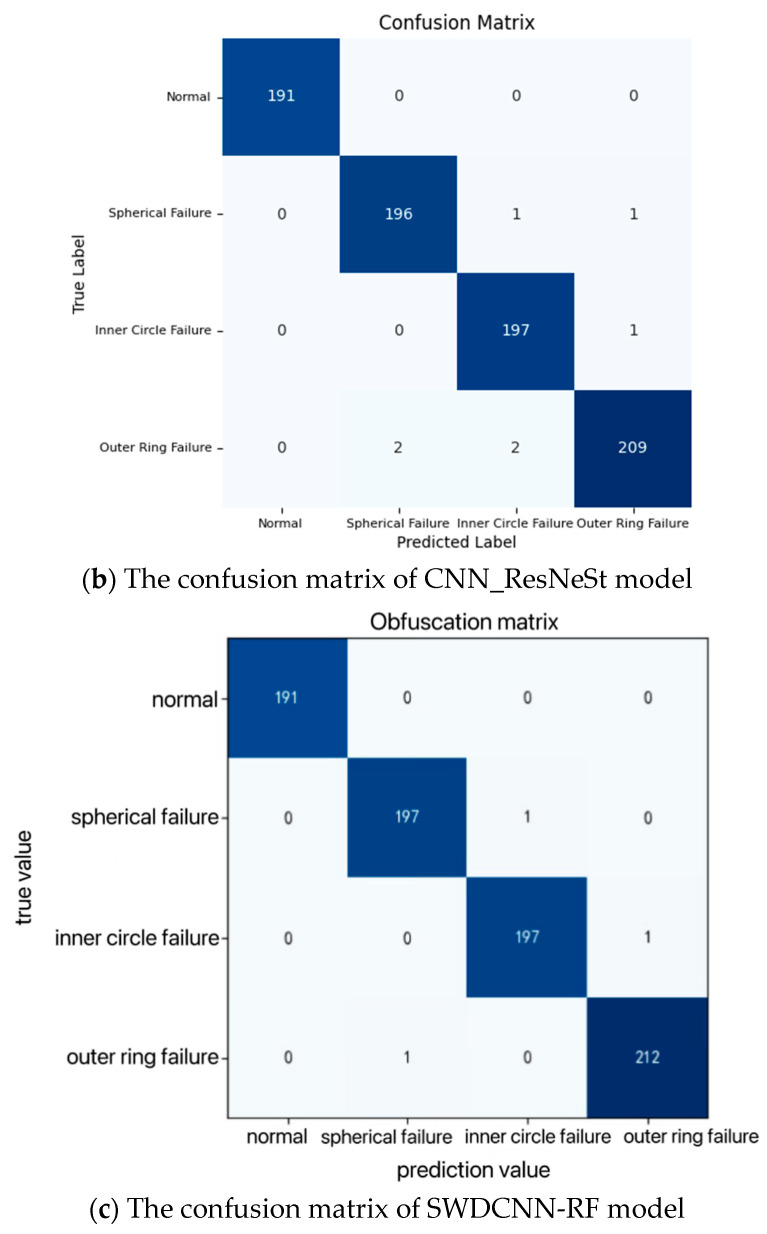
Confusion matrix.

As illustrated in Figure 13, the confusion matrix comparing the diagnostic results of three convolutional models on the test set reveals key differences. The purple color represents normal operating conditions, green indicates outer race faults, blue represents inner race faults, and yellow indicates spherical faults. The traditional WDCNN and CNN-ResNeSt models show minor errors in predicting the spherical fault mode, while the improved SWDCNN-RF model performs exceptionally well in predicting all three types of fault modes and normal operating conditions, with only minimal errors. This indicates a significant improvement in accuracy with the SWDCNN-RF model compared to the traditional WDCNN and CNN-ResNeSt. The confusion matrix visualization highlights that the WDCNN and CNN-ResNeSt models experience some degree of misclassification in detecting spherical fault modes, revealing its limitations in feature extraction and differentiating between different fault types. In contrast, the SWDCNN-RF model demonstrates nearly flawless classification across various fault modes and normal conditions, showcasing its superior generalization ability and robustness when dealing with complex vibration signal data. A deeper analysis suggests that the marked improvement in classification accuracy with the SWDCNN-RF model stems from its ability to leverage the strengths of both Wide Convolutional Neural Networks (WDCNNs) and Random Forests (RFs). This hybrid approach enhances the model’s ability to extract and differentiate critical features, resulting in higher diagnostic precision and a more reliable performance in real-world fault diagnosis scenarios.

## 4. Conclusions

To enhance the speed and accuracy of ball bearing fault recognition at varying speeds, this paper presents a ball bearing fault diagnosis model based on an improved Wide Convolutional Neural Network (WDCNN). The model was validated using the ball bearing dataset provided by Western Reserve University, leading to the following key conclusions:

(1) Improved Processing Speed: At 50 training epochs, the SWDCNN-RF model demonstrated a 38.51% faster response speed than the traditional WDCNN model and was 26.02% faster than the CNN-ResNeSt model when processing vibration signal data at mixed speeds. This significant improvement in processing speed, coupled with reduced training fluctuations, highlights the model’s ability to maintain stable and efficient performance when dealing with complex and diverse input data. This is highly valuable for real-time fault diagnosis in practical industrial applications.
(2) Enhanced Diagnostic Accuracy: The SWDCNN-RF model significantly improved the diagnostic accuracy of convolutional neural networks when processing the rolling bearing dataset of Western Reserve University, with an accuracy of 99.6%. This result indicates that the model has excellent performance in handling rolling bearing fault diagnosis tasks and can accurately identify and classify different types of faults. This high-precision diagnostic capability is of great significance for predicting and preventing equipment failures, extending equipment lifespan, and reducing maintenance costs.
(3) Strong Generalization and Robustness: The superior performance of the SWDCNN-RF model is not only reflected in speed and accuracy but also in its strong generalization ability and robustness. The model performs well under different working conditions and can effectively cope with various noise and interference factors, which makes its application prospects in industrial environments very broad. By combining the advantages of wide convolutional neural networks and random forests, the SWDCNN-RF model not only demonstrates powerful capabilities in the field of fault diagnosis but also provides an efficient and reliable solution for other similar time series analysis tasks.

## Figures and Tables

**Table 1:** Table of Output Dimensions for Each Layer.

Network Layer	Input Size	Output Size
Input layer	(1, 1024)	(1, 1024)
Convolutional layer 1	(1, 1024)	(16, 128)
Batch normalization layer 1	(16, 128)	(16, 128)
Activation Layer 1 (ReLU)	(16, 128)	(16, 128)
Pooling layer 1	(16, 128)	(16, 64)
Convolutional layer 2	(16, 64)	(32, 64)
Batch normalization layer 2	(32, 64)	(32, 64)
Activation Layer 2 (ReLU)	(32, 64)	(32, 64)
Pooling layer 2	(32, 64)	(32, 32)
Exhibition layer	(32, 32)	(1, 1024)
Dropout	(1, 1024)	(1, 1024)
Fully connected layer 1	(1, 1024)	(1, 32)
Activation Layer 3 (ReLU)	(1, 32)	(1, 32)
Fully connected layer 2 (encoder)	(1, 32)	(1, 8)
Activation Layer 4 (ReLU)	(1, 8)	(1, 8)
Fully connected layer 3 (output layer)	(1, 8)	(1, 2)
Softmax	(1, 2)	(1, 2)

**Table 2:** Parameter Table of SWDCNN-RF Model.

Num	Network Layer	Kernel Size/Stride (Neurons)	Number of Filters (Kernels)	Output Size (Width × Depth)	Zero Padding
1	1D Convolution 1	64 × 1/16 × 1	16	128 × 16	Yes
2	Batch Normalization 1	16	-	128 × 16	No
3	1D Pooling 1	2 × 1/2 × 1	-	64 × 16	No
4	1D Convolution 2	3 × 1/1 × 1	-	64 × 32	Yes
5	Batch Normalization 2	32	32	64 × 32	No
6	1D Pooling 2	2 × 1/2 × 1	-	32 × 32	No
7	Fully Connected 1	1024	-	32 × 1	No
8	Fully Connected 2 (Encoder)	32	-	8 × 1	No
9	Fully Connected 3	8	-	4 × 1	No
10	Softmax	4	-	4	No

**Table 3:** Parameter Table of WDCNN Model.

Num	Network Layer	Kernel Size/Stride (Neurons)	Number of Filters (Kernels)	Output Size (Width × Depth)	Zero Padding
1	Convolution 1	64 × 1/16 × 1	16	128 × 16	Yes
2	Pooling 1	2 × 1/2 × 1	16	64 × 16	No
3	Convolution 2	3 × 1/1 × 1	32	64 × 32	Yes
4	Pooling 2	2 × 1/2 × 1	32	32 × 32	No
5	Convolution 3	3 × 1/1 × 1	64	32 × 64	Yes
6	Pooling 3	2 × 1/2 × 1	64	16 × 64	No
7	Convolution 4	3 × 1/1 × 1	64	16 × 64	Yes
8	Pooling 4	2 × 1/2 × 1	64	8 × 64	No
9	Convolution 5	3 × 1/1 × 1	64	6 × 64	Yes
10	Pooling 5	2 × 1/2 × 1	64	3 × 64	No
11	Fully Connected	100	1	100 × 1	-
12	Softmax	10	1	10	-

**Table 4:** Parameter Table of CNN-ResNeSt Model.

Num	Network Layer	Kernel Size/Stride (Neurons)	Number of Filters (Kernels)	Output Size (Width × Depth)	Zero Padding
1	Input Layer	-	-	1000 × 2	-
2	Conv1D	3 × 1/1 × 1	32	998 × 32	No
3	Conv1D	3 × 1/1 × 1	64	996 × 64	No
4	MaxPooling1D	16 × 1	-	62 × 64	No
5	Residual Block(repeated)	3 × 1/1 × 1	64	62 × 64	Yes
-	-Conv1D(within block)	3 × 1/1 × 1	64	62 × 64	Yes
-	-Conv1D(within block)	3 × 1/1 × 1	64	62 × 64	Yes
-	Add & Activation	-	64	62 × 64	Yes
6	Conv1D	3 × 1/1 × 1	64	60 × 64	No
7	GlobalAveragePooling1D	-	-	64	-
8	Dense	-	256	256	-
9	Dropout	-	-	256	-
10	Dense(Output Layer)	-	4	4	-

## Data Availability

The data presented in this study are available on request from the corresponding author.
